# The American Medical Association

**Published:** 1907-07-20

**Authors:** 


### THE AMERICAN MEDICAL ASSOCIATION.

Some of the addresses delivered at the recent meeting of the American Medical Association throw an interesting light on the inner history of the profession in the great Republic, and the difficulties they define may well appeal to those who have similar problems to face in our own country. Among the questions discussed, prominence was given to medical education, to the relations of laboratory investigations to clinical work, and to the need for "honest drugs." To refer first to the last-mentioned, some rather startling statements were made by the President, Dr. Joseph D. Bryant. Quoting from the reports of the Board of Health of New York City, it was alleged that serious departures from the official strength were not uncommon in the case of a number even of well-recognised and frequently employed drugs. Thus of 15 samples of various preparations of aconite three had considerable excess of alkaloid, while in two the active principle was distinctively defective. An identical state of matters was reported in preparations of other potent drugs, such, for example, as belladonna, nux vomica, and opium. It must be observed that practically all of the above preparations are capable of an exact estimate of their alkaloidal values by definite chemical tests, and that therefore the errors must be attributed to carelessness or fraud in manufacture. The conclusion is inevitable that many manufacturing pharmacists do not bring to their work the care which the importance of their calling demands. The consequences, however, may be as disastrous in one case as in the other, for constancy of strength of medicinal preparations is an absolutely essential condition of safe and effective prescribing. As Dr. Bryant pointed out in his address, the medical profession is not altogether free from blame in this matter. A readiness to "try" new drugs and to prescribe proprietary preparations rather than well-established official remedies weakens the position of the competent and scientific pharmacist, and discourages the cultivation of a high degree of dispensing skill. We hold it to be a substantial interest both of the medical profession and of the public that the practice of pharmacy should be in the hands of those who have been trained, not only in technical accomplishments, but also in a high sense of duty and responsibility. And such results are only possible when the individual importance of the pharmacist as a trustworthy agent in the preparation of medicines is fully recognised.

On the relations of the laboratory worker to the physician, the American Association had an opportunity of listening to some wise counsel from Dr. James B. Herrick. He protested against the notion that the laboratory could provide a short cut to diagnosis. Its findings ought rather to be considered as additional facts to be added to those discovered by bedside observation, and a diagnosis to be secure must include the latter not less than the former. Strictly speaking, the function of the laboratory worker ceased with the announcement of the facts he had discovered, and it was no part of his duty to intrude on the fields of diagnosis and treatment. Especially is it necessary to recognise that it is only the physician, who is in immediate contact with the patient, who is competent to appreciate the full responsibility of the position; the laboratory worker is detached from the patient, and cannot therefore realise all the aspects of the case. Dr. Herrick strongly urged a more close association between the clinical pathologist and the physician. This is to be accomplished by more thorough training of the medical student in laboratory methods. These ought not to be regarded as outside the usual clinical service of the hospital ward, but should be treated as part and parcel of the general scheme of clinical investigation. Much of what was said on this point to our American *confrères* may profitably be studied by ourselves. Yet we gladly admit that within recent years a thoroughly healthy and sound teaching and practice in regard to laboratory work have distinguished the clinical instruction in our British schools of medicine. At first, as was not unnatural, the novelty of the development led to an exaggeration of its influence. Now it is recognised in its true position as a valuable servant to, but by no means the master of, the physician. It may, and often does, enlarge our category of facts, but it is not the final word apart from clinical observation and study. The physician must be trained in the presence of the patient, but he ought also to learn to increase his power and value by the resources open to him in the clinical laboratory.

